# *In silico* and *in vitro* Evaluation of Mimetic Peptides as Potential Antigen Candidates for Prophylaxis of Leishmaniosis

**DOI:** 10.3389/fchem.2020.601409

**Published:** 2021-01-15

**Authors:** Deborah Carbonera Guedes, Manuel Hospinal Santiani, Joyce Carvalho, Carlos Ricardo Soccol, João Carlos Minozzo, Ricardo Andrez Machado de Ávila, Juliana Ferreira de Moura, Eliezer Lucas Pires Ramos, Guillermo Raul Castro, Carlos Chávez-Olórtegi, Vanete Thomaz-Soccol

**Affiliations:** ^1^Programa de Pós-Graduação Strictu Sensu em Engenharia de Bioprocessos e Biotecnologia, Universidade Federal do Paraná, Curitiba, Brazil; ^2^Centro de Produção e Pesquisa de Imunobilógicos, Secretaria De Saúde do Estado do Paraná, Piraquara, Brazil; ^3^Programa de Pós Graduação em Ciência da Saúde, Universidade do Extremo Sul Catarinense, Criciúma, Brazil; ^4^Laboratorio de Nanobiomateriales, CINDEFI, Departamento de Química, Facultad de Ciencias Exactas, Universidad Nacional de La Plata (UNLP)-CONICET (CCT La Plata), La Plata, Argentina; ^5^Max Planck Laboratory for Structural Biology, Chemistry and Molecular Biophysics of Rosario (MPLbioR, UNR-MPIbpC), Partner Laboratory of the Max Planck Institute for Biophysical Chemistry (MPIbpC, MPG), Centro de Estudios Interdisciplinarios (CEI), Universidad Nacional de Rosario, Rosario, Argentina; ^6^Departamento de Bioquímica e Imunologia, Instituto de Ciência Biológicas, Universidade Federal de Minas Gerais, Belo Horizonte, Brazil

**Keywords:** mimetic peptides, cytokines, *in vitro* infection, vaccines, leishmaniasis

## Abstract

Antigen formulation is the main feature for the success of leishmaniosis diagnosis and vaccination, since the disease is caused by different parasite species that display particularities which determine their pathogenicity and virulence. It is desirable that the antigens are recognized by different antibodies and are immunogenic for almost all *Leishmania* species. To overcome this problem, we selected six potentially immunogenic peptides derived from *Leishmania* histones and parasite membrane molecules obtained by phage display or spot synthesis and entrapped in liposome structures. We used these peptides to immunize New Zealand rabbits and determine the immunogenic capacity of the chimeric antigen. The peptides induced the production of antibodies as a humoral immune response against *L. braziliensis* or *L. infantum*. Next, to evaluate the innate response to induce cellular activation, macrophages from the peptide mix-immunized rabbits were infected *in vitro* with *L. braziliensis* or *L. infantum*. The peptide mix generated the IFN-γ, IL-12, IL-4 and TGF-β that led to Th1 and Th2 cellular immune responses. Interestingly, this mix of peptides also induced high expression of iNOS. These results suggest that the mix of peptides derived from histone and parasites membrane molecules was able to mimic parasites proteins and induce cytokines important to CD4+ T cell Th1 and Th2 differentiation and effector molecule to control the parasite infection. Finally, this peptide induced an immune balance that is important to prevent immunopathological disorders, inflammatory reactions, and control the parasite infection.

## Introduction

Despite all the advances in the field of immunization and different strategies to identify new antigenic molecules, there is still no antigen capable of inducing *Leishmania* spp. control and protecting individuals against leishmaniosis. Moreover, the large number of *Leishmania* species, responsible for cutaneous and visceral forms, and the differences among them make the diagnosis of these pathologies harder (Maroof et al., 2012). Thus, it is essential to search for new technologies to develop antigen candidates for diagnosis or a vaccine to induce the control of these diseases in individuals who reside in at risk regions (De Brito et al., 2018).

Given this scenario, an attractive alternative is peptide-based antigens that use epitopes of immunogenic proteins, which can stimulate a long-lasting immune response against the pathogen. This approach is a promising strategy, since it could even promote protection against *Leishmania*, it is a potent therapeutic tool to treat the disease (Skwarczynski and Toth, 2016; De Brito et al., 2018), could be applied in diagnosis (Link et al., 2017), and used to develop antigens to stimulate cellular immunity for potential use in vaccine protocols (Hamrouni et al., 2020). Furthermore, peptides are easier to produce and show greater stability. These antigens are produced by chemical synthesis, reducing problems with biological contamination. Antigens can be characterized as chemical molecules, similar to classical drugs, their production is reproducible, simple, cost-effective and fast, and low cost to scale-up (Joshi et al., 2014). Concerning immunity, these antigens can be customizing to generate specific responses and can be combined to design multi-epitopes or multi-specific antigens to target different *Leishmania* species or immunogenic molecules from different stages of the parasite life cycle. Despite all the advantages, this approach has some challenges, such as enhancing the immunogenicity of the peptides. One of the strategies to overcome this challenge is to design a multi-epitope-based antigen, which consists of incorporating multiple epitopes that allows for better coverage of natural pathogen antigen diversity (Moyle and Toth, 2013; De Brito et al., 2018).

The effectivity of a vaccine to promote a long-lasting cell mediated immune response also depends on the molecules used as antigens in the different production approaches. A high diversity of virulent *Leishmania* molecules have been tested as antigens, including gp63 (glycoprotein leishmaniolysin), SLA (soluble *Leishmania* antigen), LPG (lipophosphoglycan), histones, and several other purified antigens (Khamesipour et al., 2006; Olivier et al., 2012; Chamakh-Ayari et al., 2014; Martínez Salazar et al., 2014; Link et al., 2017).

Among these molecules, histones are potential candidates against leishmaniosis, since they constitute structural proteins that are important in the organization and regulation of the parasite genes. There are four major *Leishmania* histone classes –H2A, H2B, H3, and H4– that are basic component units of chromatin, the nucleosome (Requena et al., 2000). These molecules are highly conserved antigens, produced by several *Leishmania* species, which are non-secreted, but are able to induce an intense immune response (Santarem et al., 2007). These proteins are released during the infection process after the elimination of intracellular amastigotes by active macrophages. Moreover, they can modulate the host immune response, since they do not suffer selective pressure by the host immunity, unlike surface secreted proteins of *Leishmania* (Chang et al., 2003).

The identification and selection of an epitope are crucial stages to develop a peptide-based antigen. It is necessary to map the whole protein of interest to identify suitable sequences that can induce a strong and permanent cellular immunity response against *Leishmania* parasites. These epitopes can be identified *in silico* and analyzed by bioinformatics tools that focus on T and B cell epitopes prediction, identification of conserved *Leishmania* species sequences, and location of the sequence on the protein quaternary conformation (Herrera-Najera et al., 2009; Freitas et al., 2016). The second approach is *in vitro* analysis of the epitopes by biotechnological and biochemical tools, such as phage display and spot synthesis techniques (Pini et al., 2005; Rhaiem and Houimel, 2016).

Our research group has been working in these areas to improve and develop diagnosis and prophylaxis techniques for the different neglected tropical diseases (NTDs). Some of these previous works have shown great promise regarding the evaluation of mimetic peptides as antigens for leishmaniosis diagnosis and vaccines (Seger, 2014; Link et al., 2017; Guedes et al., 2019). Based on previously obtained results with three mimetic peptides selected by phage display and chemically synthesized as soluble molecules, their potential as antigens was evaluated in skin tests. The peptides, individually (PA1, PA2, and PA3) or in a mix (PA1,2,3-Mix), were tested on an animal model (*Cavia porcellus*) immunized with dead *L. amazonensis* or *L. braziliensis* and compared with the standard skin test antigen. The results showed that the peptides, individually or in a mix, promoted induration reactions 48 and 72 h after inoculation (Guedes et al., 2019). These results indicate that these peptides can recruit and maintain a desired immune response and that they can be applied as antigens for immune prophylaxis purposes. Based on these findings, this work proposed the investigation of new active peptides derived from histone proteins of *Leishmania* spp. though the production of a chimeric molecule with the three peptides previously selected by our group. The biological activity of this chimeric molecule was investigated to verify its potential to activate a satisfactory immune response for diagnosis or as a candidate vaccine.

## Materials and Methods

**Figure 1:**
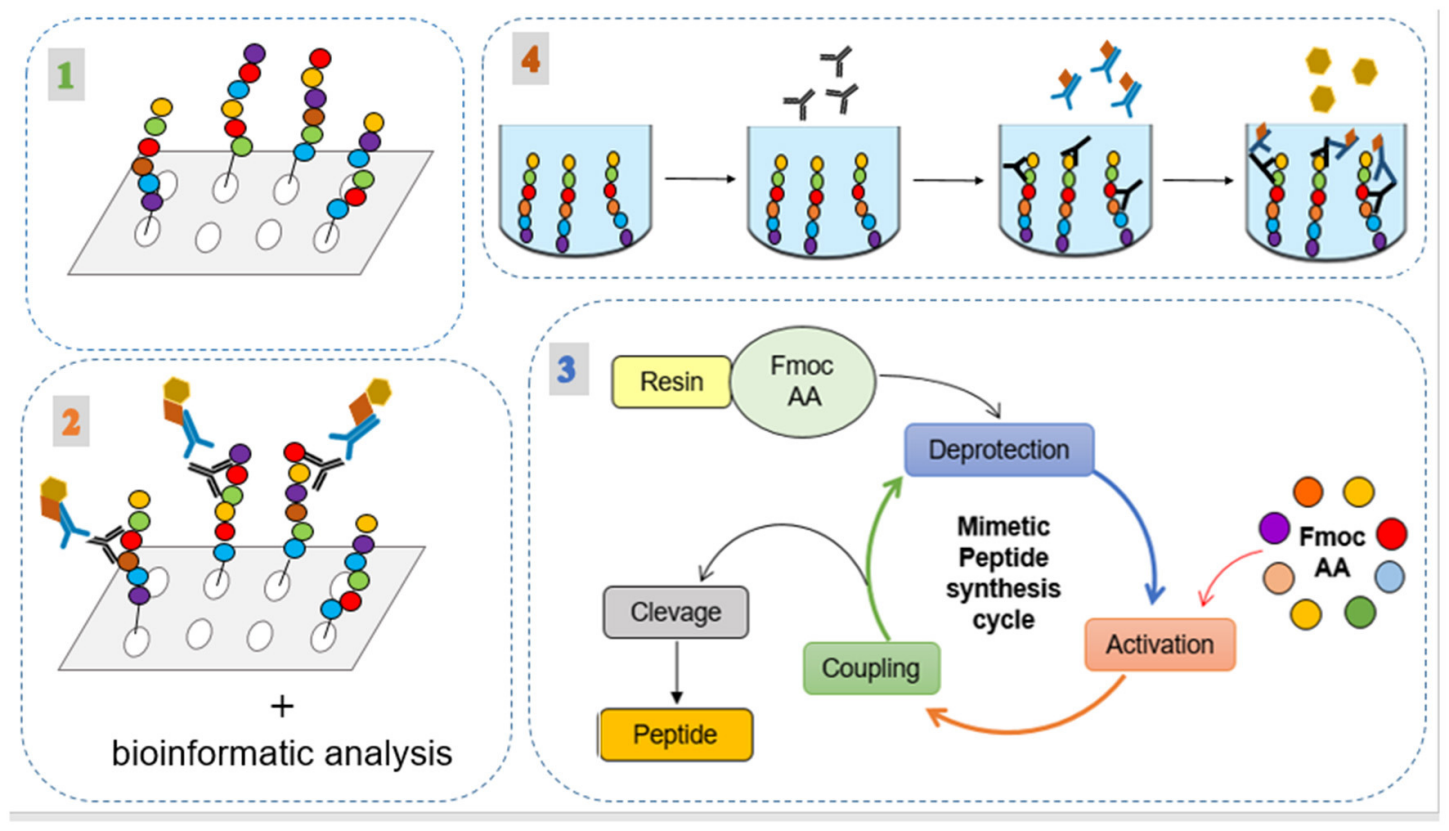
Experimental processes for the selection, synthesis, and evaluation of the peptides. (1) Spot synthesis of the intracellular immunogenic proteins from Leishmania spp.; (2) Immunodetection assay of reactive spots; (3) their analysis by bioinformatic tools; and (4) application of the peptides in indirect ELISA method using human serum.

**Figure 2:**
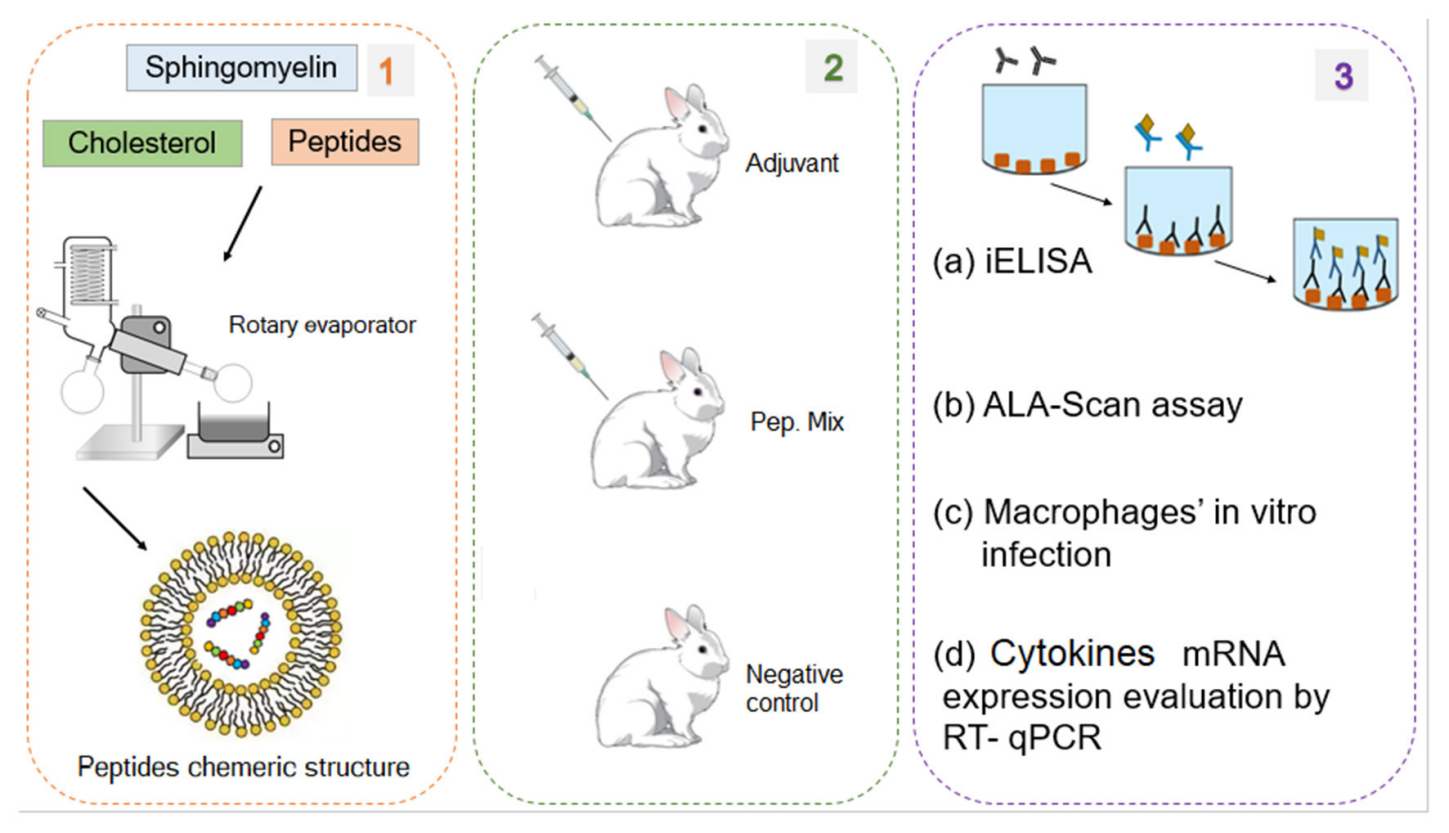
Experimental processes for the production and evaluation of the peptide chimeric molecules. (1) Peptides encapsulation in liposomes. (2) Rabbits immunization with mix of six peptides (chimeric molecule) to produce anti-peptide polyclonal antibodies. (3) Evaluation of the peptides immunogenic capacity to induce immune response: (a) ELISA assay to evaluate IgG antibody production profile; (b) Evaluation of ALA scanning peptides with rabbit anti-peptide polyclonal antibodies; (c) *in vitro* experimental infection of macrophages from rabbits immunized with peptides; (d) Evaluation of cytokines mRNA expression profile by RT-qPCR.

The method used in this work consists of two main steps illustrated in Figures 1, 2. The first involved the selection, synthesis, and evaluation of peptides. The second included experimental procedures to evaluate the immunogenicity of these peptides by analyzing humoral and macrophages immune responses. Regarding the first step, seven intracellular immunogenic proteins from *Leishmania* spp. (histone classes H2A, H2B, H3 and H4, and HP1) were selected and spot synthesized, followed by the selection of reactive peptides by immunodetection assay (Figure 1, step 1). The reactive spots were analyzed by bioinformatics tools to evaluate their chemical and structural characteristics to verify protein regions that manifest epitope-like features (Figure 1, step 2). The selected peptides were chemically synthesized (Figure 1, step 3) and encapsulated in liposome structures (Figure 2, step 1), with the other three previously selected peptides, to formulate the peptide-based antigen (see section Results). In sequence, the peptide-based antigen was used to immunize a group of New Zealand rabbits (Figure 2, step 2) and *in vitro* analyses were performed to evaluate the capacity of the peptide-based antigen to stimulate humoral and macrophage responses against *L. braziliensis* or for *L. infantum* (Figure 2, step 3). Other analyses were performed to verify the peptide characteristics that contributed to their selection (Figure 2, step 3).

All applicable international, national, and institutional guidelines for the care and use of animals were followed. The study was approved by the Research Ethics Committee of the Federal University of Parana (process no. 23075.085350/2015-54 and 107/11).

### Peptide Selection

The proteins selected for this study were immunogenic intracellular molecules derived from *L. amazonensis* and *L. braziliensis* histones. The sequences of histones were accessed on the UniProt database and identified as HP1 (UniProt accession number: Q9NL78), HP2 (Q9NL77), HP3 (Q9BMY8), HP4 (A4H9W0), HP5 (A4HBV1), HP6 (O44009), and HP7 (A4HNK4). The homology of the protein sequences among *Leishmania* sp. (*L. amazonensis, L braziliensis* and *L. infantum*) were verified by EMBOSS Needle pairwise sequence alignment (Rice et al., 2000).

The seven selected protein sequences were spot synthesized by overlapping pentadecapeptides, with an offset of three amino acids, scanning aimed at selecting and evaluating the reactivity of epitopes on these sequences. The spot synthesis was performed on a cellulose membrane with fluorenylmethyloxycarbonyl (Fmoc) protection using an automated spot peptide synthesizer (Intavis Bioanalytical Instruments, Nattermannallee, Germany) (Frank, 2002). For the immunodetection assay to select and evaluate the reactive spots, the cellulose membrane was firstly blocked, for non-specific binding, by overnight agitation with 3% (w/v) casein and 0.5% (w/v) sucrose dissolved in TBS-T (0.1% Tween 20 (v/v) in TBS) at 4°C. Afterwards, the membrane was washed with 0.1% TBS-T for 10 min under agitation, and probed, for 90 min at 37°C, with patients serum containing antibodies for *L. braziliensis* or *L. infantum*, or negative control diluted 1:100 in blocking buffer (3% (w/v) casein, 0.5% (w/v) sucrose, and 0.1% TBS-T). The membrane was washed again and incubated with biotin-labeled secondary antibody (1:30,000) diluted in blocking buffer for 60 min at 37°C, followed by an incubation step with streptavidin (1:10,000) diluted in blocking buffer for 60 min at 37°C. After two washes, positive spots were visualized by electrochemiluminescence (ECL^TM^ system).

To complement the selection of reactive peptides, the protein sequences were analyzed using bioinformatics tools such as Peptide 2.0 (https://www.peptide2.com/main_about.php), IPC (Kozlowski, 2016) and PepCalc (https://pepcalc.com/) to evaluate characteristics like molecular weight, isoelectric point, net charge, and hydrophobicity. Other tools, such as Epitopia server (Rubinstein et al., 2009), ABCpred (Saha and Raghava, 2006), and IEDB Analysis Resource (Vita et al., 2015), were used to verify protein regions that manifest epitope-like characteristics. Additionally, the 3D structure of proteins was obtained by a homology-modeling server, SwissModel (Guex and Peitsch, 1997), using the histones X-ray diffraction structure as templates and the predicted epitopes were analyzed by Swiss-Pdb Viewer (Guex and Peitsch, 1997). Analyses of the 3D structures of the proteins were performed to evaluate where the reactive peptides (their sequences) were located on the protein structure, which contributed to verifying whether they are more exposed or internal. Finally, the peptide sequences were BLASTed against *Leishmania* spp. protein sequences, using TriTrypDB to analyze the similarities between them (Aslett et al., 2010).

### Chemical Synthesis of Selected Peptides

The peptides selected by immunodetection assay and analyzed by bioinformatics tools were chemically synthesized according standard protocol by Fmoc strategy (9-fluorenylmethyloxycarbonyl) using a resin as insoluble solid support (Merrifield, 1969) with a MultiPep RS automated peptide synthesizer (Intavis Bioanalytical Instruments, Nattermannallee, Germany). After the final synthesis cycle, the peptides were released from the resin by trifluoracetic acid treatment, filtered and precipitated with cold ethyl ether, yielding the peptides. After centrifugation, the ether was discarded and peptides were lyophilized, weighed, dissolved in ultrapure water, and stored at −20°C until the next step.

To reduce the number of peptides selected by the bioinformatics tools after chemical synthesis, they were tested by indirect ELISA (iELISA) for humoral response using serum from human patients with anti-*L. braziliensis* or anti-*L. infantum* antibodies. The aim was to determine whether the peptides induce a mimicked response to the parasite.

### Reactive Peptides Encapsulation in Liposome

The immunogenic peptides (selected in this work by spot synthesis) plus three peptides previously selected by our research group (selection determined by phage display and hypersensitivity reaction named P1, P2, and P3 for detail, see Link et al., 2017; Guedes et al., 2019), were encapsulated in liposome according to Toledo-Machado et al. (2015), producing the peptide-based antigen. Briefly, the encapsulated peptides were produced by dissolving sphingomyelin (25 mg) and cholesterol (6.5 mg) in 5 mL solution containing methanol and chloroform (1:2). The solvent was removed by flash evaporation on a rotatory evaporator at 37°C and dried for 80 min under reduced pressure. Next, an aqueous phase containing the six peptides (500 μg of each peptide) diluted in 3 mL of PBS pH 7.4 was added to lipid film composing the liposome structures with the six peptides encapsulated. To dislodge and retrieve the liposomes they were treated three times with ultrasonic vibration for 20 s. To remove no encapsulated peptides, the liposome suspension was washed twice by centrifugation (10 min, 8,000 g at 4°C) and resuspended in PBS pH 7.4. After washing, the liposomes were lyophilized and stored at 4°C.

### Polyclonal Antibody Production

To produce polyclonal antibodies, five adult female New Zealand White rabbits (NZW), weighing 2.1–2.6 kg were used. The rabbits were housed in single cages in a standard animal room (20°C and 55% humidity) and fed a balanced diet and water ad libitum. The rabbits were divided in three groups: Group 1, immunized with the adjuvant (aluminum hydroxide); Group 2, immunized with the peptides mix entrapped in liposome; and Group 3, no immunization (uninfected group). For the first immunization (day 0), Group 1 was submitted to intramuscular injection with 1 mL of aluminum hydroxide; Group 2 was submitted to intramuscular injection with 500 μg/rabbit (Melo et al., 2020) of entrapped peptides mix dissolved in PBS buffer (pH 7.4) and 1 mL of aluminum hydroxide (adjuvant); and Group 3 (negative control) was not immunized. The other immunizations were performed under the same conditions on days 15, 30, 45, 60, and 90 after the first immunization (a.f.i.). On day 90, the rabbits were bled, euthanized and macrophages were collected for the *in vitro* assay.

Blood samples for polyclonal antibody reactivity evaluation were obtained before every immunization (days 0, 15, 30, 45, and 60), by vein puncture from the marginal ear vein of the rabbits and transferred to vacutainer blood collection tubes. For cytokines evaluation, blood samples were collected at 48 and 72 h a.f.i. (day 0) and transferred to vacutainer blood collection tubes containing 200 μL of TRIzol. After each sample collection, they were centrifuged at 448 x g, for 10 min, at room temperature, resuspended in red blood cell (RBC) lysis buffer pH 7.3 (89.9 g of NH_4_Cl; 10 g of KHCO_3_; 2 mL of EDTA 0.5M), incubated for 5 min and centrifuged for 2 min, 1,008 x g at room temperature. The supernatant was discarded, and the pellet was resuspended in PBS, centrifuged for 2 min, 1,008 x at room temperature and resuspended in 400 μL of TRIzol. The samples were stored at −80°C.

### Anti-IgG Antibody Production Profile

An indirect ELISA assay was performed to evaluate the profile of the polyclonal antibody reactivity obtained during the rabbits' immunization period. The microtiter plates were coated with *L. braziliensis* or *L. infantum* protein extracts at a concentration of 1 μg/well prepared according to Link et al. (2017). Rabbit sera, with anti-peptide polyclonal antibodies, and the second antibody (anti-rabbit IgG (whole molecule) peroxidase–Sigma Aldrich) were respectively diluted 1:200 and 1:7,500 in plates coated with *L. braziliensis* protein extract, and 1:100 and 1:10,000 in plates coated with *L. infantum* protein extract.

### *In vitro* Experimental Infection of Macrophages From Rabbits Immunized With the Peptides Chimeric

#### Macrophages *in vitro* Infection With *L. braziliensis* and *L. infantum* Promastigote Forms

After 90 days post immunization, the rabbits were euthanized, and the macrophages were obtained by inoculation of 120 mL cold PBS buffer 1x (pH 7.4) in the peritoneal cavity. The PBS was collected and transferred to a tube and maintained on ice. The material collected was centrifuged at 1,800 x g, for 10 min at 4°C. The pellet was washed with PBS buffer 1x (pH 7.4), and centrifuged (1,800 x g, for 10 min at 4°C). The pellet was resuspended in RPMI 1640 + 20% FBS + 10% penicillin-streptomycin and counted in a Neubauer chamber. For *in vitro* infection, the parasites were obtained from cultures of *L. braziliensis* and *L. infantum* separately cultivated in biphasic brain heart infusion medium until stationary phage (5 days). The parasites were harvested from culture media, centrifuged twice with PBS for 15 min, 1,000 x g at 10°C, resuspended in RPMI 1640 + 20% FBS + 10% penicillin-streptomycin, and counted in a Neubauer chamber. For the *in vitro* infection, 500 μL of parasite suspension were added to each well containing the adhered macrophage cells (1 × 10^5^ cells/well). The parasites were added in a proportion of 15:1 (parasites: cells) (1.5 × 10^6^ parasites/well) and were incubated at 37°C and 5% CO_2_. Next, the RNA was extracted 48 and 72 h post infection (p.i.) to analyze cytokines, inducible nitric oxide synthase (iNOS) expression, and the parasite load.

#### Evaluation of Cytokines and iNOS mRNA Expression by Real Time PCR (RT-qPCR)

A reverse transcriptase and quantitative real time PCR (RT-qPCR) was performed to assess the expression of mRNAs of cytokines and iNOS in blood samples, collected between 48 and 72 h a.f.i. in rabbits, and the peritoneal macrophages from immunized rabbits (90 days a.f.i.) after the *in vitro* infection.

Total RNA of the samples was extracted using RNeasy Mini Kit (QIAGEN®), according to the manufacturer's instructions. To remove contaminating genomic DNA from RNA samples, a TURBO DNase-free kit (Invitrogen®) was used. The samples were quantified by spectophometers, and 150 ng of isolated RNA was reverse transcribed to cDNA using a First Strand cDNA Synthesis kit (Thermo Scientific®) with oligos dT_15_. Complementary DNA was mixed with 10 pmol of each gene-specific marker and 2.5 μL of SYBR Green PCR Master Mix (Applied Biosystem). The mRNA expression of cytokines and iNOS was assessed using StepOnePlus Real-Time PCR System (Thermo Scientific). Data analysis was performed by the Livak method (2^−ΔΔCt^) (Livak and Schmittgen, 2001) using the housekeeping genes glyceraldehyde 3-phosphate dehydrogenase (GAPDH) and beta-actin (ACTB) to normalize mRNA expression.

#### DNA Extraction From *in vitro* Macrophage Infection

Total DNA of samples was extracted using the Dynabeads™ DNA DIRECT™ Universal kit (Invitogen, Vilnius, Lituania), according to the manufacturer's instructions. The DNA eluate was stored at −20°C until its use in qPCR analysis.

#### Parasite Load by Quantitative Real-Time PCR (qPCR)

The qPCR reactions were performed with 5 μL DNA; 2X KAPA Probe Fast ABI Prism® from KapaBiosystem; 200 nM DpolyAF (5′ GACDGTGAATTACAGGHTGC 3′) and DpolyAR (5′ ATACTTGCAGCAGCACATCG 3′) primers and 100 nM DpolyAS probe (5′FAM- TCACTTGCACHCCAGATK -NFQ-MGB3′) specific for the DNA polymerase A of *Leishmania* sp. Cycling conditions were a first step at 95°C for 10 min, followed by 40 cycles at 94°C for 30 sec and 58°C for 30 min. The amplifications were performed in a QuantStudio™ 3 (Applied Biosystems, USA). Standard calibration curves were constructed by serial dilution of DNA extracted samples of *L. braziliensis* from 10 to 0.001 ng.

### Statistics Analyses

The results were presented as means ± SD. Differences in antibody titers, parasite load and cytokine production were assessed by a two-way ANOVA followed by a Tukey HSD comparison test. All statistical analysis was performed using GraphPad Prism version 7.0 software. A *p*-value of <0.05 was considered statistically significant.

## Results

### Peptide Selection: *In silico* and *in vitro* Analysis

First, the proteins HP1 and HP2 from *L. amazonensis* were compared with the protein HP6 from *L. braziliensis*; all three proteins correspond to the histone subunit H3. The analysis showed that between the proteins HP1 and HP6 similarity is 84.6% and identity is 94.6%. For proteins HP2 and HP6, similarity is 85.4% and identity is 95.4%. Consequently, the similarity and identity of the protein sequences were analyzed with *L. infantum* histone subunit fractions. The parameters of similarity and identity were not analyzed for HP3, because the protein sequence of histone subunit fraction H1 was not found for *L. braziliensis* and *L. infantum*.

### Immunodetection

**Figure 3:**
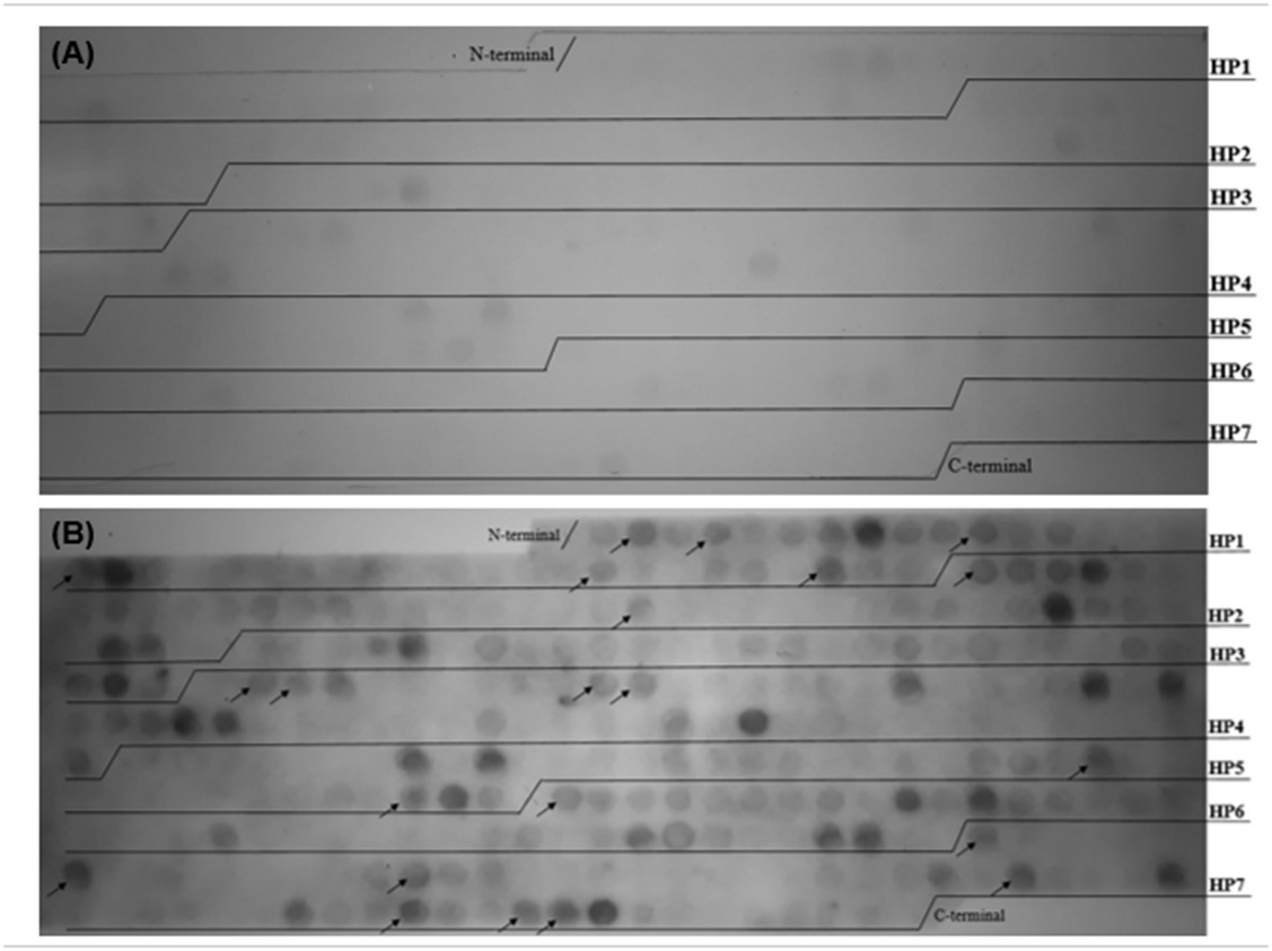
Histone proteins sequences were spot synthesized by overlapping pentadecapeptides offset by three amino acids aimed at selecting and evaluating the reactivity of epitopes on these sequences. Reactivity of peptides originated from histone proteins of *Leishmania* spp. with anti-*L. braziliensis* immunoglobulins G. **(A)** Reactivity of peptides with healthy patient serum without anti-*Leishmania* spp. IgG. **(B)** Reactive spots immunodetected by patient serum with antibodies for *L. braziliensis* and not cross-reacted with healthy patient serum (without anti-*Leishmania* spp. IgG). The arrows indicate the 22 peptides selected by the immunoassay.

The spot synthesis of the seven histone sequences (HP1, HP2, HP3, HP4, HP5, HP6, and HP7) generated 302 spots, such that each spot was a different pentadecapeptide. The reactive epitopes were immunodetected by patient serum with anti-*L. braziliensis* antibodies. Twenty-two spots were selected, derived from almost all seven histone sequences analyzed. The 22 spots were named P_H_2, P_H_4, P_H_11, P_H_17, P_H_31, P_H_37, P_H_40, P_H_61, P_H_109, P_H_110, P_H_118, P_H_119, P_H_190, P_H_202, P_H_205, P_H_245, P_H_251, P_H_260, P_H_276, P_H_290, P_H_293, and P_H_294. The membrane was tested with serum from volunteer healthy patients, without antibodies for *Leishmania* spp., to verify that these reactions were specific (Figure 3).

### *In silico* Analysis of Peptide Sequences

Bioinformatics tools (Peptide 2.0, IPC, PepCalc) enabled the analysis of peptide sequences and those with a hydrophobicity lower than 55% were selected for the next steps of the study (Table 1). In fact, hydrophobic peptides are insoluble hindering their use. The *Leishmania* spp. histone proteins sequences used in this study were 3D modeled using the X-ray diffraction structure of histones as templates. The structures of HP1, HP2 and HP6 were constructed by homology with histone H3. The tridimensional structure HP4 was constructed with histone H2B; histone H2A was used for HP5; and for HP7, the template was constructed by homology with histone H4.

**Table 1:** *In silico* analysis of reactive peptides.

**Peptides**	**Molecular weight (g/mol)**	**Iso-electric Point**	**Net charge**	**Hydrophobicity (%)**	**Peptide source**	**CD4 T cell immunogenicity prediction**	**T cell class I pMHC immunogenicity prediction**
P_H_2^*^	1649.89	9.66	4	20	H3.1	71.85	−0.27
P_H_4^*^	1535.79	9.76	5	26.67	H3.1	51.16	−1.18
P_H_11^*^	1864.19	11.37	3	40	H3.1	63.99	0.61
P_H_17^*^	1747.03	9.1	1.9	40	H3.1	45.92	−0.02
P_H_31^*^	1599.83	3.29	−2.1	53.33	H3.1	46.20	−0.16
P_H_37^*^	1779.14	10.55	3	46.67	H3.1	59.58	−0.10
P_H_40^*^	1750.04	11.55	4	26.67	H3.2	60.96	0.06
P_H_61^*^	1727.96	10.36	2	33.33	H3.1	54.26	−0.29
P_H_109^*^	1627.88	5.21	−0.1	53.33	H2B	55.89	−0.11
P_H_110^*^	1659.97	5.21	−0.1	53.33	H2B	46.26	−0.47
P_H_118	1825.12	9.95	1.2	60	H2B	47.46	0.15
P_H_119	1865.16	6.59	0	60	H2B	55.34	0.32
P_H_190^*^	1807.24	12.14	4.1	53.33	H2A	46.71	0.01
P_H_202^*^	1610.94	9.84	6.1	20	H2A	60.08	−0.87
P_H_205^*^	1766.04	11.55	4	20	H3	72.93	−0.12
P_H_245	1605.95	5.83	−0.1	66.67	H4	72.28	0.34
P_H_251^*^	1523.69	10.47	1.1	46.67	H4	56.31	−0.34
P_H_260^*^	1827.16	11.15	2.2	40	H4	43.00	−0.24
P_H_276^*^	1713	10.66	5	26.67	H4	84.88	−1.10
P_H_290^*^	1882.12	4.79	−1	40	H4	42.78	0.17
P_H_293^*^	1719.87	3.96	−2.1	33.33	H4	46.25	0.24
P_H_294^*^	1776.92	4.57	−1.1	26.67	H4	53.46	0.15

The evaluated parameters are mass weight, iso-electric point, net charge, and hydrophobicity. The (

* *) indicates the peptides that present hydrophobicity lower than 55%*.

**Figure 4:**
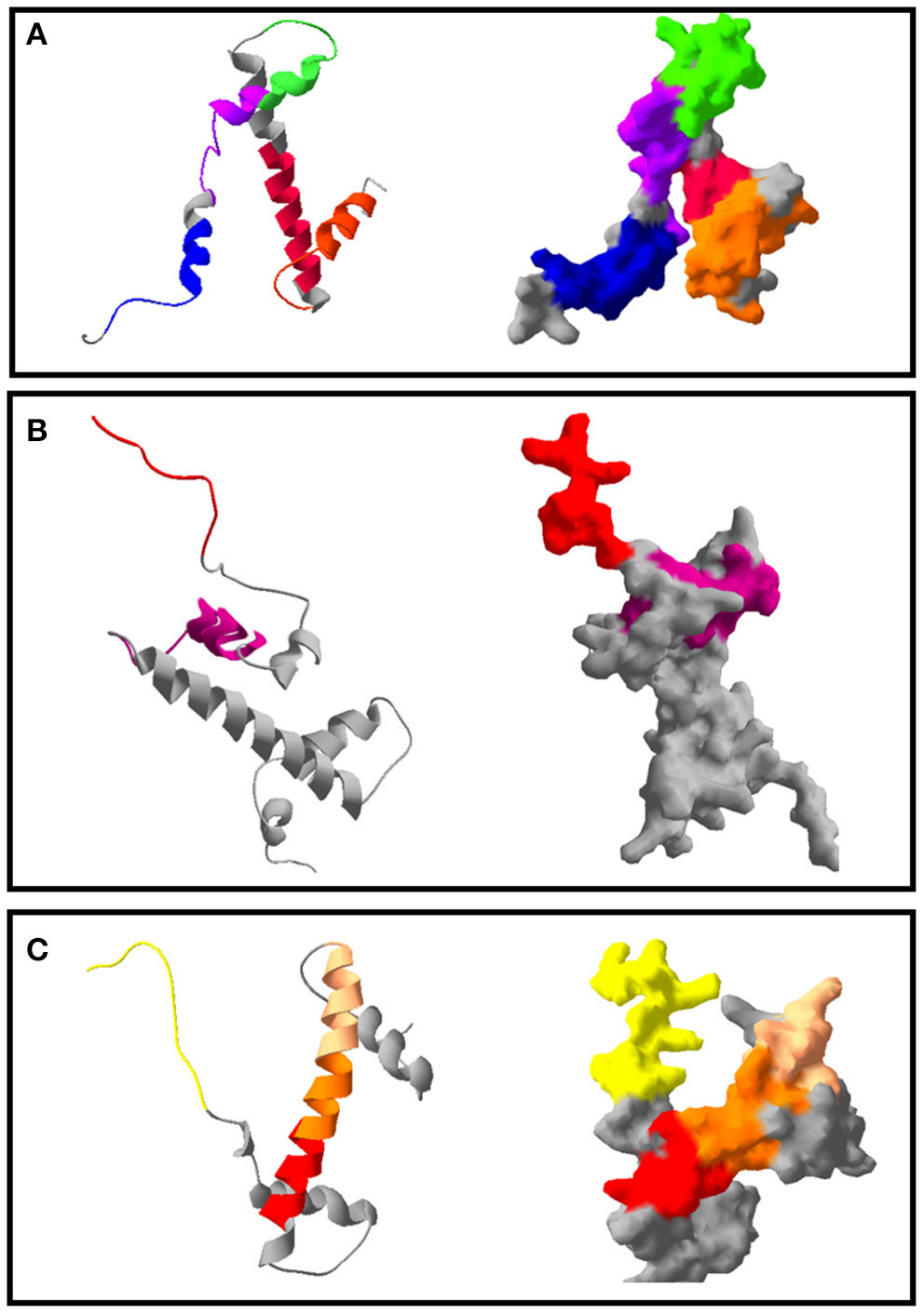
Tridimensional model of *Leishmania* spp. histones and the position of the immunodominant peptides selected. The colors indicate the different epitope regions of this sequence, which were immunodetected by anti-*L. braziliensis* IgG on the Spot Synthesis membrane. **(A)** Ribbon format and solid surface format of the protein HP1. In blue: P_H_11; in purple: P_H_17; in pink: P_H_31; in orange: P_H_37 and in green: P_H_61. **(B)** Ribbon and solid surface format of the protein HP5. In pink: P_H_190; in red: P_H_202. **(C)** Ribbon and solid surface format of the protein HP7. In yellow: P_H_276; in red: P_H_290; in orange: P_H_293; in light pink: P_H_294.

**Figure 5:**
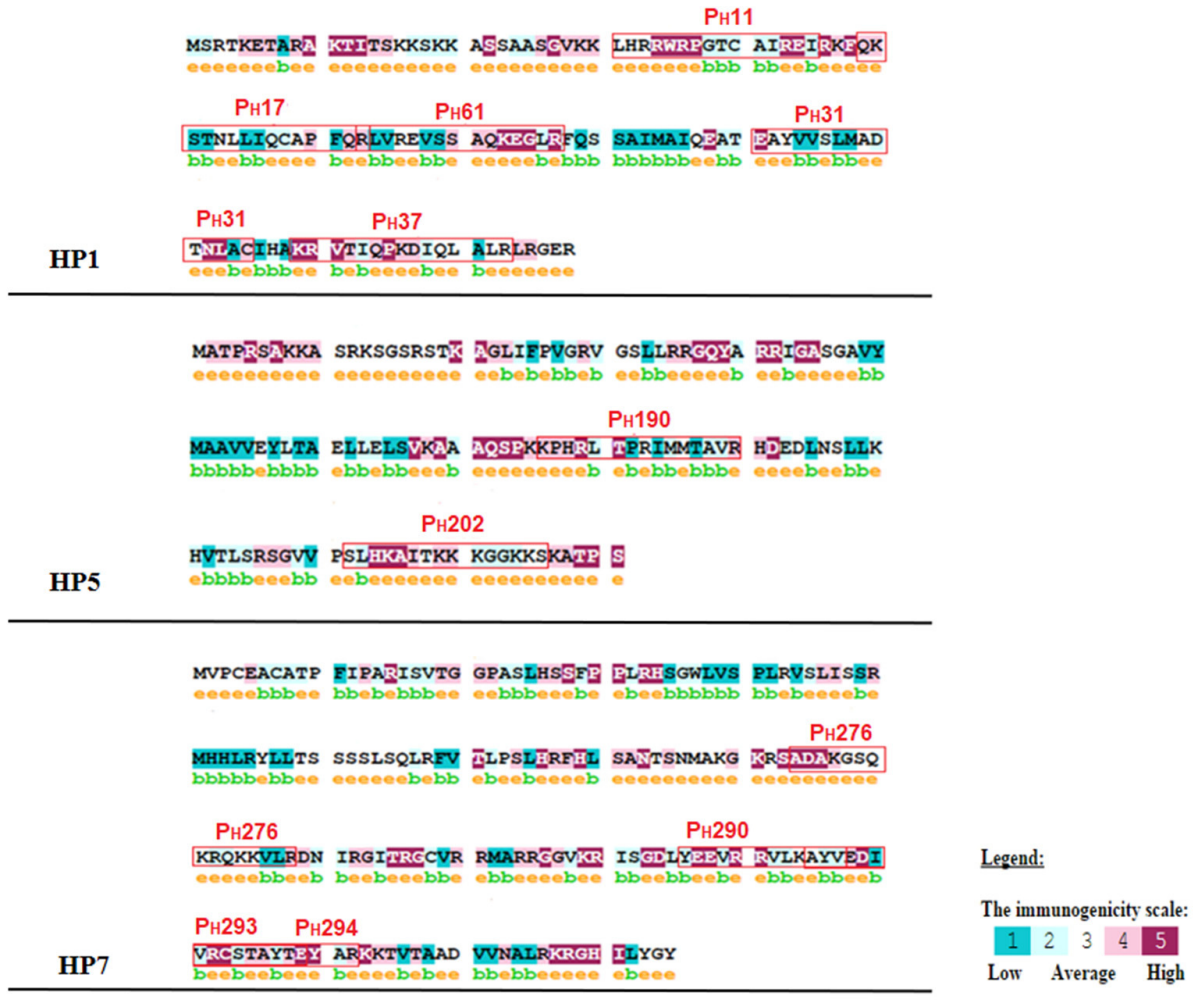
Immunogenicity analysis of the reactive peptides. The letter e, in yellow and above amino acids, indicates a predicted exposed residue. The letter b, in green and above amino acids, indicates a predicted buried residue. A color scale indicates the immunogenicity of each amino acid, which is present in the figure. The immunogenicity was classified as 1 for low and 5 for high for each amino acid.

According to these alignments, the tridimensional structures of the proteins HP1, HP2, HP5, and HP7 were constructed (Figure 4). The alignment of HP1 and HP2 sequences was made with the same template from histone H3, so these two proteins have the same 3D structure. After constructing the tridimensional structures by homology modeling, the peptides were located on the structures using the Swiss-PDB Viewer program. For HP1, the reactive peptides P_H_11, P_H_17, P_H_31, P_H_37, and P_H_61 were highlighted on the histone protein structure. The same was performed for HP5 with peptides P_H_190 and P_H_202, and for HP7 with peptides P_H_276, P_H_290, P_H_293, and P_H_294. After these analyses, the reactive peptides were evaluated by Epitopia server (Figure 5) and IEDB Analysis Resource to verify their immunogenic characteristics (Table 1). After all these analyses, the amino acid sequences of the selected peptides were BLASTed against *Leismania* spp. histone proteins sequences to verify the identity between them.

### Chemical Synthesis of Selected Peptides and Their Evaluation by Indirect ELISA

The synthesis of peptides resulted in 2.5 mg of each soluble molecule, which were resuspended in 1 mL of ultrapure water. Each peptide was tested as an antigen in an iELISA assay at concentrations of 0.25, 0.5, and 1 μg/mL.

The results showed that all the peptides showed immunoreactivity in patient serum for anti-*L. braziliensis* (*n* = 13) and for anti- *L. infantum* (*n* = 10) antibodies. In the iELISA test for patient serum with anti- *L. braziliensis* antibodies, the best results were observed for peptides P_H_31, and P_H_293, which reached an absorbance 2.5-fold higher than the positive and negative controls. These results were observed for the peptides tested under the conditions of 0.25 μg/mL of antigen, 1:100 of serum and 1:7,500 of conjugate in the case of P_H_31; and of 0.5 μg/mL of antigen, 1:200 of serum and 1:10,000 of conjugate for P_H_293 (Table 2). For anti- *L. infantum* antibodies, the best results were observed for P_H_202 and P_H_293 under the same conditions of 0.5 μg/mL of antigen, 1:200 of serum, 1:10,000 of conjugate, where the absorbance was 2.5 times higher from positive and negative controls (Table 2). According to these test results, the peptides P_H_31, P_H_202, and P_H_293 were selected and analyzed in biological assays as antigen candidates.

**Table 2:** Indirect ELISA reaction parameters standardized for peptides PH31, PH202, and PH293.

	**Peptide (μg/mL)^a^**	**Serum^b^ (*N* = 23)**	**Second antibody^c^**	**Absorbance^d^**
**Anti–*****L. braziliensis*** **IgG**				
P_H_31	0.25	1:100	1:7,500	0.132
P_H_293	0.5	1:200	1:10,000	0.120
**Anti–*****L. infantum*** **IgG**				
P_H_202	0.5	1:200	1:10,000	0.123
P_H_293	0.5	1:200	1:10,000	0.137

a Antigen (peptide) concentration on indirect ELISA assay;

b Patients serum concentration;

c Second antibody concentration;

d *The absorbance of negative serum samples was 0.05*.

### Production and Evaluation of Peptides Chimeric Structure

**Figure 6:**
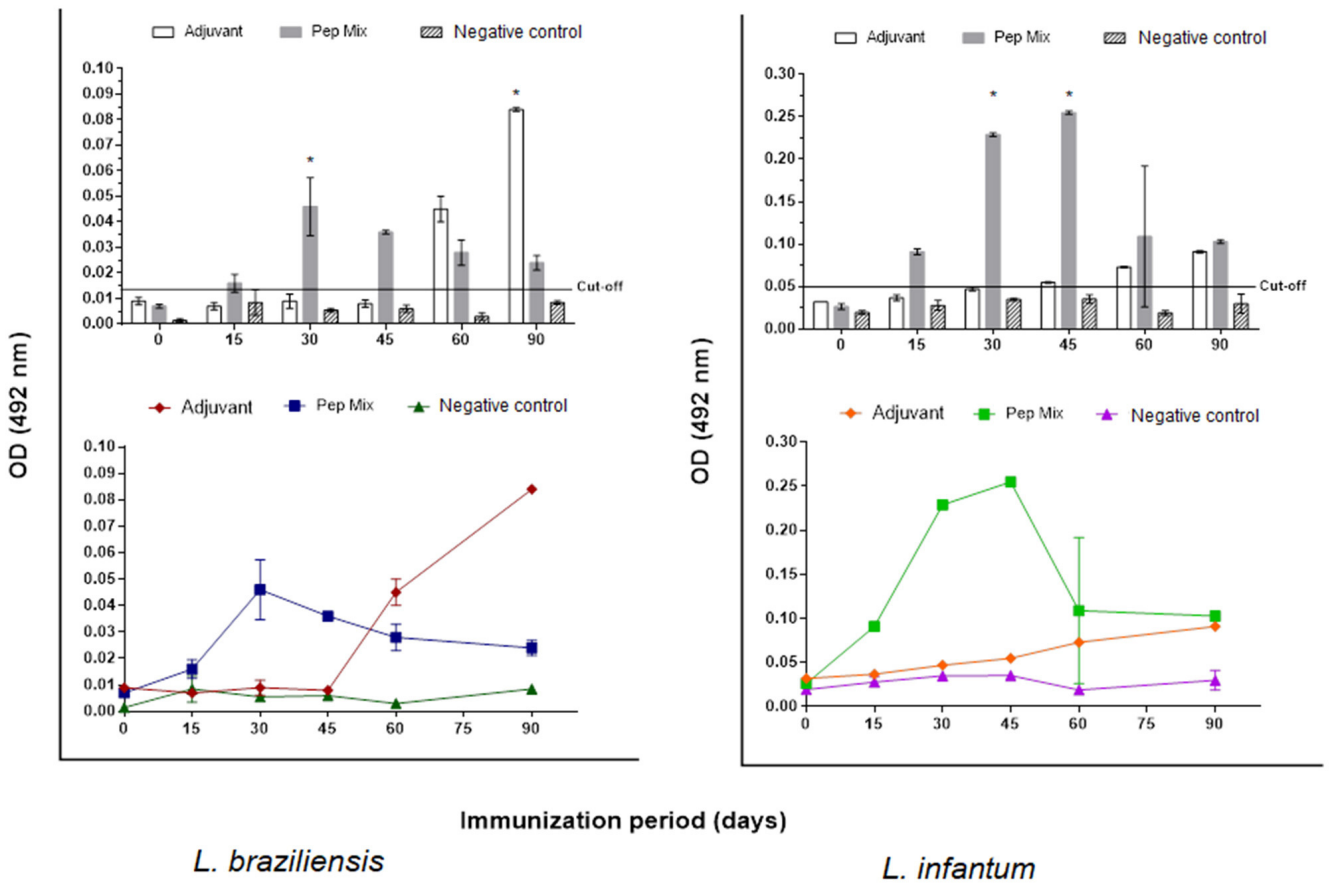
Reactivity of anti-peptide polyclonal antibodies against *L. braziliensis* and/or *L. infantum* protein extract antigen tested by indirect ELISA assay. Kinetics of IgG production for 90 days in: Group 1: rabbits immunized with aluminum hydroxide (adjuvant). Group 2: rabbits immunized with peptides mix entrapped in liposomes. Group 3: non immunized rabbits (negative control). The red line indicates the cut-off value 0.014 for *L. braziliensis* and 0.050 L. infantum. Statistical test two-way ANOVA followed by a Tukey HSD comparison test *P < 0.05.

The encapsulation of peptides resulted in five samples of chimeric molecules at a concentration of 500 μg/mL. To formulate the antigen for animal immunization, each sample was resuspended in 1 mL of adjuvant resulting in a final concentration of 500 μg/mL. The antibody response with the entrapped mix of peptides, evaluated by iELISA test showed that producing anti-peptides polyclonal antibodies 15 days a.f.i. was feasible. This production was detected by all the kinetics evaluated (until 90 days a.f.i.) for both *L. braziliensis* and *L. infantum* protein extracted antigen (Figure 6).

### Cytokines and Effector Molecule Induction by the Chimeric Molecules

**Figure 7:**
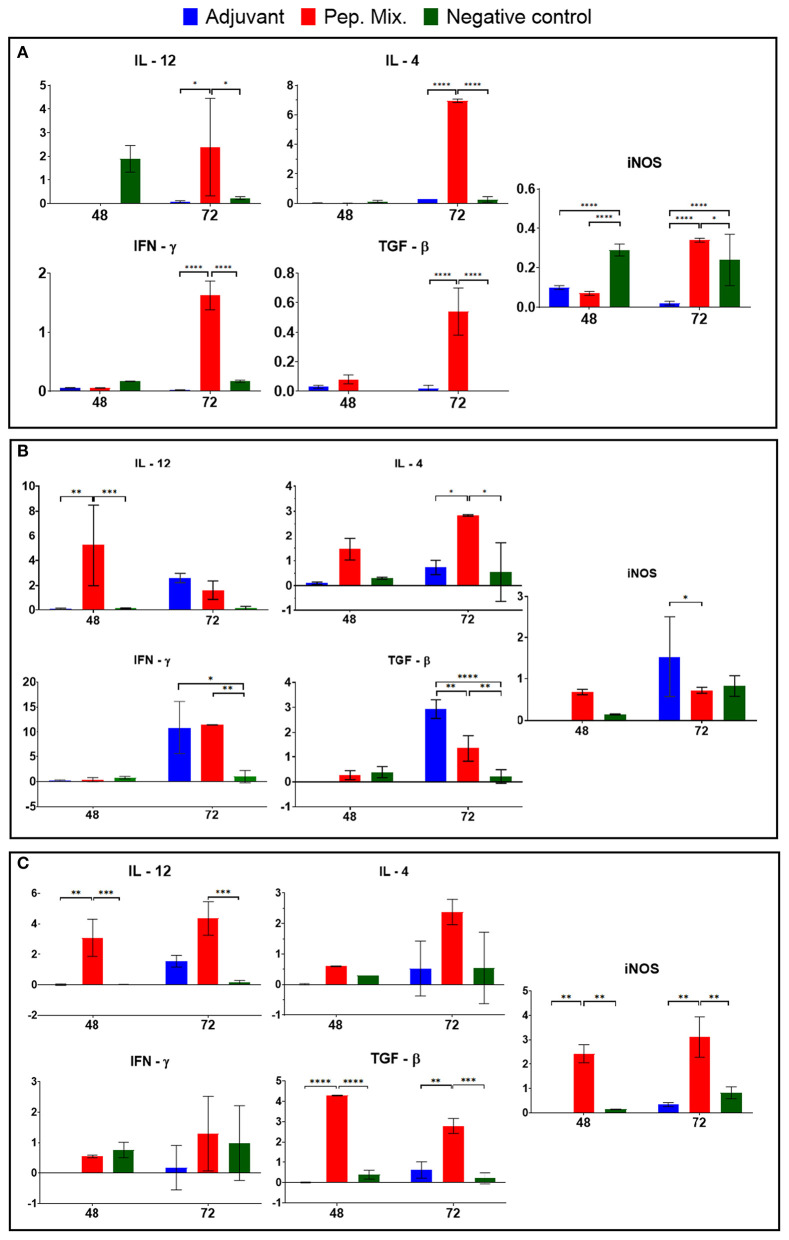
Cytokine (IL-12, IFNγ, IL-4, and TGF-β) and iNOS mRNA expression were evaluated in blood samples **(A)** and macrophages from rabbits immunized with adjuvant, or peptides mix, and non-immunized (negative control) after infection by *L. braziliensis* **(B)** and *L. infantum* **(C)**. All the results are presented as the mean ± SD. Statistical test two-way ANOVA followed by a Tukey HSD comparison test *P < 0.05; **P < 0.01; ***P < 0.001; ****P < 0.0001, a comparison was with negative control.

To understand how the immunization could activate the immune response in the different groups, the expression of inflammatory Th-1 inductor cytokines (IL-12, IFN-γ), Th2 inductor cytokine (IL-4), regulatory cytokine (IL-10) and effector molecule (nitric oxide) inductor (iNOS) was measured 48 and 72 h a.f.i. in the blood of the rabbits. The peptide mix (Group 2) induced high expression of all the molecules evaluated 72 h a.f.i., compared with the other groups; this was significant, with a *P*-value > 0.01. High expression of IL-12 and iNOS was observed in the control group 48 h a.f.i. Group 1 (adjuvant) only induced iNOS expression 48 h a.f.i. (Figure 7A).

After the collection of peritoneal macrophages, from rabbits previously immunized with the peptides mix, infection in culture with *L. braziliensis* was performed and analyzed after 48 and 72 h. High expression was observed in the rabbits immunized with the peptide mix, showing a significant difference compared with the other groups (*P*-value > 0.01) at 48 h p.i., which decreased by 72 h p.i. In addition, the IFN-γ expression was increased in this group 72 h p.i. and showed a significant difference in relation to the negative control group, while iNOS expression was the same at both times evaluated. The peptide mix group (Group 2) also induced IL-4 and TGF-β 72 h p.i. and showed a significant difference in relation with the other groups (*P*-value > 0.1). The adjuvant group (Group 1) showed increased expression of all the cytokines evaluated and iNOS 72 h p.i., while the control group only induced the expression of iNOS 72 h p.i. (Figure 7B).

Peritoneal macrophages were also infected in culture with *L. infantum* and high expression of IL-12, TGF-β and iNOS was observed 48 h p.i., showing a significant difference compared with the other groups (*P*-value > 0.01), while IL-4 and IFN-γ also increased 72 h p.i. in the peptide mix group (Group 2). No significant expression was observed in the other groups after infection by this parasite (Figure 7C).

**Figure 8:**
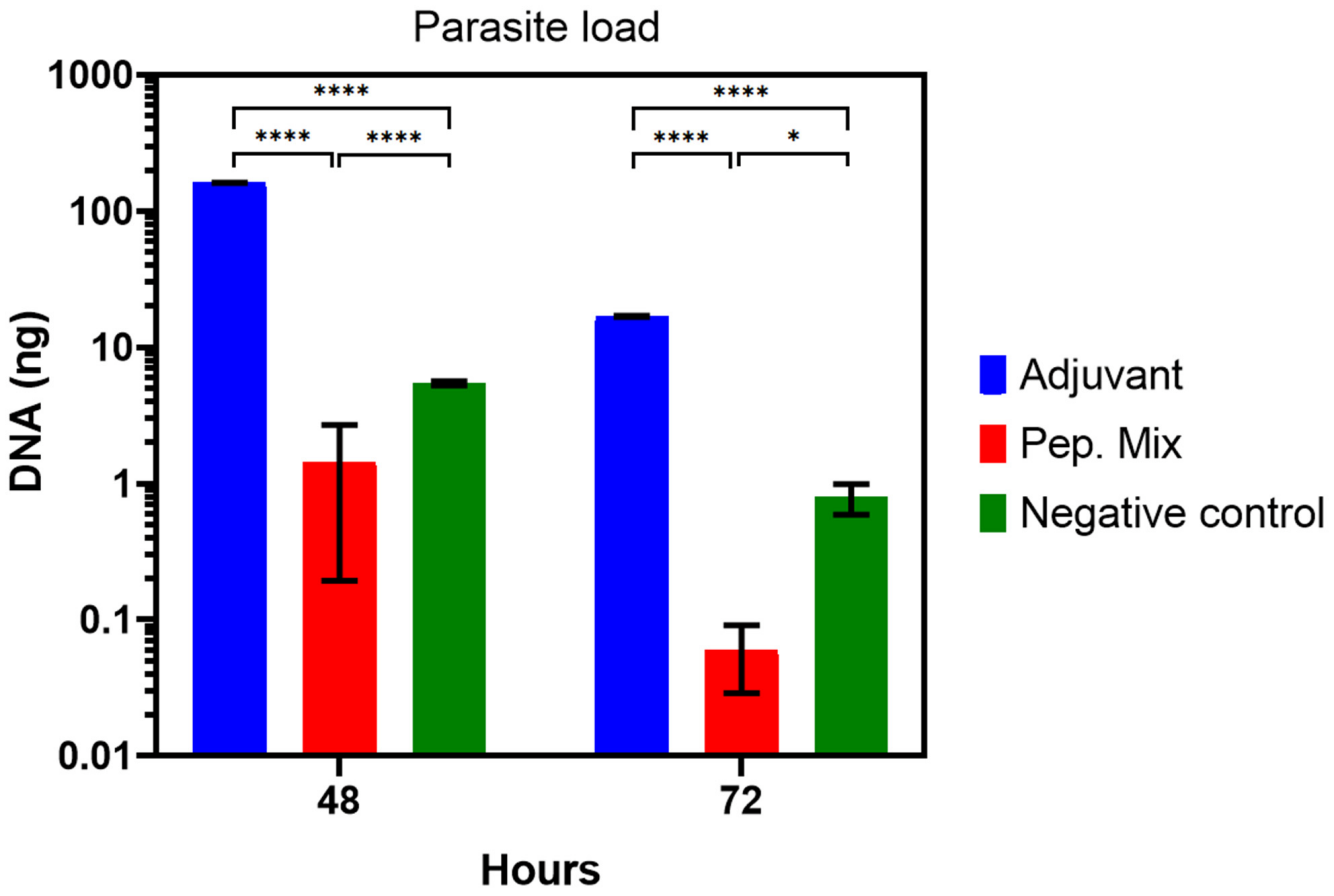
Parasite load from *in vitro* infection with *Leishmania* of macrophages from rabbits immunized with adjuvant, or peptides mix, and non-immunized (negative control), 48 or 72 h post-infection. All the results are presented as the mean ± SD. Statistical test two-way ANOVA followed by a Tukey HSD comparison test *P < 0.05; **P < 0.01; ***P < 0.001, ****P < 0.0001.

Regarding parasite load, a significant reduction in the load of *Leishmania* was observed in macrophages from rabbits immunized with the peptide mix in relation to the negative control and adjuvant group at 48 h (*P*-value > 0.0001) and 72 h (*P*-value > 0.1) (Figure 8). The parasitism in macrophages from the adjuvant group showed an increase compared with the negative control group, which is probably related to a non-specific cellular activation caused by this compound that favors *Leishmania* infection in these cells. Finally, these results indicate an activation of the macrophages by the peptide mix, facilitating the neutralization of *Leishmania* amastigotes.

## Discussion

*Leishmania* spp. are intracellular parasites that have complex mechanisms to survive and multiply inside the host system of phagocytic mononuclear cells. The production of antibodies and/or cellular immunity during host infection is induced because most *Leishmania* antigens are recognized by the host system. However, the immune response to some of these antigens does not protect the host. In other cases, it may contribute to intensifying the pathological response, due to cross-reaction with the host's biomolecules (Handman, 2001). In addition, some studies report that the capacity to respond to *Leishmania* antigens varies from individual to individual. Thus, the diagnosis of our vaccine against leishmaniosis requires different antigens (polyvalent) capable of inducing a protective immune response in most of the population and which can be produced in large scale (Mukherjee et al., 2013).

To achieve higher antigenicity, proteins were selected to target antigenic epitopes and then constructing a chimeric molecule that has greater antigenicity. Thus, seven protein sequences of *Leishmania* spp. histones were selected, which are one of the intracellular immunogenic molecules from this parasite. The core histone of eukaryotic species comprises two paired dimers H2A/H2B and H3/H4 and a linker histone H1, which constitute some of the most well-conserved molecules among these organisms. For these characteristics, these molecules are good protein candidates to screen conserved immunogenic epitopes among the different *Leishmania* spp. (Baharia et al., 2014). The histone protein sequences derived from *L. amazonensis* were named HP1, HP2, and HP3, while those derived from *L. braziliensis* were named HP4, HP5, HP6, and HP7. The proteins HP1, HP2, and HP6 correspond to histone fraction type H3, protein HP3 corresponds to H1, HP4 to H2B; HP5 to H2A, and protein HP7 corresponds to H4. These sequences were evaluated according to the similarity and identity among them. The results showed that the sequences have more than 84% similarity and 90% identity. When compared with *L. infantum* histones, the score varies from 52 to 92% similarity and 49 to 82% identity. The results indicate good similarity and identity between the *Leishmania* species, which is desirable to enable the selection of epitopes with amino acid sequences that can be recognized by different species from the parasite and formulate an antigen capable of inducing an immune response for different *Leishmania* species. In the last few years, several studies have described the isolation and application of different biomolecules that present protective activity and are potentially good antigen candidates for a leishmaniosis vaccine (Singh and Sundar, 2012; De Brito et al., 2018; Thomaz-Soccol et al., 2018). These antigens are originated from proteases and other molecules actively secreted by the parasites or from membrane and intracellular proteins. Among these molecules are histones, which are highly conserved antigens produced by *Leishmania* spp. and even though they are not secreted by the parasites they can induce a potent immune response. These antigens are released during infection after the elimination of intracellular amastigotes by the active macrophage, or by the spontaneous cystolysis of amastigotes inside the infected cells (Carrión et al., 2009). Moreover, these antigens are capable of modulating the host immune response because they do not suffer from its immune selective pressure, unlike surface and secreted proteins (Chang et al., 2003).

The antigenicity of the core histones (H2B, H2A, H3, and H4) have been reported in studies as being recognized by sera from cutaneous (CL) and mucocutaneous leishmaniosis (MCL) human patients and by canine visceral leishmaniosis (CVL) (Soto et al., 1999; Meddeb-Garnaoui et al., 2010; Souza et al., 2013). The protein H2A has been described as the most antigenic core histone and one which can also be recognized by sera from VL human patients (Passos et al., 2005). Other studies report the analysis of histone H2A, H3, and H4 immunization against CL by applying the histones individually, in cocktails, or genetically fused to a plasmid. BALB/c mice genetically immunized with the individual histones showed a delay in the development of the lesion, and immunization with the plasmids encoding the histone and the cocktails provided protection against *L. major* (Carrion et al., 2008). A study by Iborra et al. (2004) evaluated the prophylactic activity of *L. infantum* histones in an animal model for cutaneous leishmaniasis, and reported that the animals immunized with a mixture of the four plasmids encoding the histones H2A, H2B, H3, and H4 developed a specific Th1 response associated with histone-specific production of IFN-γ. Carneiro et al. (2012) also analyzed the immune protection conferred by nucleosomal histones of *L. infantum* in murine model infected with *L. braziliensis*, and concluded that histone are potential targets for vaccine formulation against *L. braziliensis* since they showed significant inhibition activity for the disease.

To select the most reactive and antigenic peptides from spot synthesized histones, this study evaluated whether the reactive peptides were located in immunogenic regions of the proteins and whether they were able to manifest epitope-like characteristics. The results showed that all the peptides were located in regions with an immunogenicity score higher than 70% (Table 1) and the majority of the amino acids of the peptides were considered to be predicted exposed residues (Figure 6). In the last *in silico* analysis, the peptide sequences were BLASTed against *Leishmania* spp. protein to evaluate the identity rate between them and to analyze whether the peptide sequences were homologous to *Leishmania* histones. The results showed that all the peptides sequences analyzed presented more than 90% identity with histone proteins from different *Leishmania* spp., mainly for *L. braziliensis* and *L. infantum*. The results also confirmed the identity of each peptide sequence with its own original histone sequence.

All these *in silico* analyses resulted in the selection of the peptides PH11, PH17, PH31, PH37, and PH61 (histone H3), PH190 and PH202 (histone H2A), and PH276, PH290, PH293, and PH294 (histone H4), which presented a response score for *in silico* CD4 analysis ranging from 42.78 to 84.9 (Table 1). The results of the iELISA study showed that some peptides did not show a satisfactory difference between serum positivity and negativity. It may not be able to induce a mimetic response like the parasite. Thus, the peptides selected according to these specifications were PH31, PH202, and PH293. Peptide PH31 showed good reactivity against anti-*L. braziliensis* antibodies, as did PH202 for anti-*L. infantum* antibodies. Peptide PH293 showed good reactivity against anti-*L. braziliensis* antibodies and for *L. infantum* IgG. We have shown here that the humoral response (optical density) was low compared with serological tests, where the differences between positive and negative are 8 to 10 times greater. However, the peptides were able to mimic the antigen. The milestone in *Leishmania* histone antigenicity studies was the screening of cDNA expression libraries using sera from infected dogs. The first report on a specific immune response against histones during infection was the identification of histone H2A from *L. infantum* by immunoscreening with CVL serum (Soto et al., 1992). Later, a study by Soto et al. (1994) reported the isolation of a cDNA clone coding *L. infantum* histone H3 by a strong immunoreaction with VL sera. In a subsequent study, the authors demonstrated that the histones H2B and H4 from *L. infantum* were also recognized by VL sera (Soto et al., 1999). Moreover, a study by Lakhal et al. (2012) reported the diagnostic performance of a crude *Leishmania* histone used as antigen in an ELISA assay in which the reactivity was accessed by sera from VL patients. The results showed the ability of this antigen to discriminate between VL cases and healthy controls. These studies report the ability and capability of histone proteins at being recognized by humoral immune molecules, which affirms their antigenicity potential for use as antigen molecules in diagnosis. This also corroborates our results for the anti-peptide histone humoral response tests, in which the reactivity of the peptides against human patient serum with anti- *L. braziliensis* or anti- *L. infantum* antibodies was verified. The peptides did not achieve the expected yield, as shown by *Leishmania* protein extract or recombinant proteins. This is because, in comparison to peptides, the protein extracts and recombinant proteins offer a greater variety of reactive epitopes, which consequently can recruit a greater immune response. This hypothesis reinforces the need for testing different combinations of molecules/peptides designed for anti-*Leishmania* vaccines to induce a strong immune response (Alonso and Soto, 2014).

After this step, we selected three reactive peptides (P_H_31, P_H_202, and P_H_293) and added three more previously selected by our research group (Thomaz-Soccol et al., 2015; Link et al., 2017; Guedes et al., 2019), which were then encapsulated in liposome. This small vesicle structure was selected to encapsulate the peptides due to its properties of biocompatibility, low toxicity, size, and hydrophobic and hydrophilic character. Moreover, liposome structures avoid decomposition of the entrapped molecules and release them at designed targets, which make them promising systems for drug delivery (Akbarzadeh et al., 2013; Alavi et al., 2017). The antibody response with the entrapped mix of peptides, evaluated by ELISA test, showed that producing anti-peptide polyclonal antibodies 15 days after the first immunization was feasible and this production was detected by all the kinetics evaluated for both *L. braziliensis* and *L. infantum* protein extract antigen (Figure 6). In both situations, a characteristic curve of antibody response kinetics with plateau phase between 30 and 45 days of immunization was observed. The first 30 days of immunization characterize the primary immune response, where immature B cells are stimulated by the antigen and become active, which induces a more specific antibody for the antigen. With repeated infection (45 days), the secondary immune response is induced when the same antigen stimulates the memory B cells leading to the production of greater quantities of specific antibodies than observed in the primary response (Abbas et al., 2015).

The group immunized only with adjuvant (aluminum hydroxide) presented anti-*Leishmania* immune response, which confirms its capacity for use as an antigen. Aluminum-based adjuvants are widely used throughout the world, and among the variants, aluminum hydroxide is the chemical most commonly used as an adjuvant. An important function of aluminum hydroxide is stimulating T cells activation and the expression of co-stimulators on antigen-presenting cells (APCs) (Abbas et al., 2015). Aluminum hydroxide mechanisms of action include depot formation, which facilitate the continued release of antigens, the formation of particulate structures promoting antigen phagocytes by macrophages and B cells, and inflammation induction that results in the activation and recruitment of macrophages (Mutiso et al., 2010; He et al., 2015). Thus, aluminum-based adjuvants can help boost the humoral immunity response by providing Th2 cells and the injection of this adjuvant can result in the priming and persistence of Th2 cells producing IL-4, IL-5, and IL-10 (Awate et al., 2013; Beck et al., 2018).

Although the previous analyses were performed by selecting biomolecules that stimulate immune response type B, some tests were performed to evaluate the cytokine profile stimulated by these peptides. These evaluations of cellular immune response sought to verify whether the peptides were capable of inducing a profile of cytokines that can lead to their application as antigens for vaccines. A protocol of *in vitro* infection was developed to identify the pattern of cytokines expressed during the immunization period and to verify whether differentiated expression of these cytokines occurred to promote a cellular immune response generated by these peptides. The results showed that the peptides induced high expression of iNOS, IL-12, and IFN-γ, 72 h after first immunization, although there were also increases in the IL-4 and TGF-β expression. This means that the peptide mix was able to induce cellular immune response in rabbits with a cytokine profile that stimulated T helper 1 (Th1) and T helper 2 (Th2) responses, but the principal information is that this immunization induced the increase of iNOS, the main NO inductor. The expression of iNOS is induced by IFN-γ in macrophages and its activation contributes to controlling the death or replication of intracellular pathogens. High-levels of iNOS are associated with the adaptive phase of immune response and its function of co-factor can contribute to the activation of IL-12 and IFN-γ in natural killer (NK) cells (Bogdan, 2015). Although transforming growth factor–beta (TGF-β) is related to the regulatory functions of the immune system, it is known that multifunctional cytokines are implicated in a variety of biological processes by enhancing cellular proliferation, activation, and stimulating cytokines of effector Th17 cells. Th17 cells show an inflammatory profile with the recruitment of neutrophils. These cells are important at the onset of infection since they can recruit more cells to the infection site and favor control of the infection (Oh and Li, 2013; Okamura et al., 2015).

The differentiation of Th1 cells is promoted mainly by IL-12 and IFN-γ and occurs in response to pathogens that activate dendritic cells, macrophages and NK cells. This profile of cytokines stimulates phagocytosis, oxidative burst and intracellular pathogen killing, regulates the expression of major histocompatibility complex (MHC), classes I and II, and thus stimulates antigen presentation to T cells (Spelberg and Junior, 2001; Abbas et al., 2015; Cortés et al., 2017). Th2 differentiation is promoted by IL-4, which stimulates high titers of antibody production and activates B cell proliferation. Moreover, Th2 plays an important role in the inflammatory process by activating mast cells and eosinophils (Spelberg and Junior, 2001; Cortés et al., 2017). Both subclasses of CD4^+^ cells are important and desired for host defense against different infectious pathogens.

The quantification of interleukins expressed by macrophages *in vitro* infected with *L. braziliensis* or *L. infantum*, showed that the peptide mix was capable of inducing a cytokine profile, which presented an increase in all the cytokines tested compared with the control group. Our data also revealed that the levels of IL-12, IFN-γ and IL-4 mRNA expression by macrophages infected with *L. braziliensis* or *L. infantum* suggests the ability of these peptides to induce Th1 and Th2 polarization that can lead to a balanced response, no inflammatory pathologies, and protective immune response. According to the literature, the host immunity to a parasite is determined by a suitable Th1 response characterized by IL-12 and IFN-γ production, and induction of iNOS in infected macrophages, which contributes to the control of parasite proliferation (Costa et al., 2002). Regarding the use of biomolecules as vaccine candidates, some studies reported an increase in the levels of IL-12 and IFN-γ in animals immunized with ribosomal protein and infected with *L. infantum* and *L. amazonensis* (Chávez-Fumagalli et al., 2010). Martins et al. (2019) tested the immunogenicity of specific protein from *Leishmania* against *L. major* and *L. braziliensis* infection, and reported that the vaccination induced a Th1 response characterized by the production of IL-12 and IFN-γ.

Although immunity against *Leishmania* is well-known and defined as complex due to the mechanisms used by the parasite to survive in the host immune system. It is well-documented that Th1 response is responsible for inducing resistance to leishmaniosis through the production of inflammatory cytokines, such as IL-12 and IFN-γ, leading to the activation of macrophages and the killing of parasites. On the other hand, susceptibility to infection is related to Th2 development and IL-4 cytokine production, which leads to parasite resistance and replication (Chávez-Fumagalli et al., 2010).

The performance of immune response in immunopathology and the immunoprotection of leishmaniosis remain a paradox. For example, some studies report that, even though the Th1 response induces inflammatory cytokine production and plays a crucial role in the immunoprotection of leishmaniosis, their excessive production can lead to severe immunopathology in the disease (Sacks and Noben-Trauth, 2002; Martin and Leibovich, 2005; Nylén and Eidsmo, 2012). However, apart from inducing the persistence of the parasite at the site of injection, the Th2 response is able to induce anti-inflammatory cytokine production at lower levels, which can mitigate inflammatory reactions and accelerate the healing process (Nylén and Eidsmo, 2012; Pasparakis et al., 2014). These studies suggest that a balance between pro- and anti-inflammatory cytokines is essential, and desirable, to prevent immunopathological disorders and inflammatory reactions and can control the infection. This fact reinforces and corroborates our results that the peptide mix was able to induce the production of a cytokine profile that leads to a Th1 and Th2 immune response. In addition, the parasite load showed a reduction in the parasite. The next step of this study should be to evaluate the peptides in an *in vivo* model.

## Conclusions

It was possible to encapsulate the peptides in the liposomes permitting the use of these molecules to entrap the peptides and delivering them for recognition as immunogenic epitopes by the immune mechanisms, while generating an appropriate immune response against these peptides. This indicates their ability to induce humoral immune response through the production of antibodies capable of reacting against *L. braziliensis* and *L. infantum*. They also demonstrate the ability to generate cellular immune response through the expression of cytokines that induce Th1 and Th2 polarization of T CD4+ cells, which leads to a balanced response, preventing immunopathological disorders and inflammatory reactions. Most of all, the peptides can lead to the expression of iNOS, the pivotal inductor of the effector molecule NO that controls parasite infection. Finally, these results suggest that the peptides can mimic the parasites proteins that present an important role in the host-parasite interaction, such as histones, and are targets to generate immunity against the parasites. In summary the mix of mimetic peptides tested in this work, by *in vitro* infection, demonstrated a satisfactory development for use as a potential candidate for leishmaniosis vaccines.

## Data Availability Statement

The original contributions presented in the study are included in the article/supplementary material, further inquiries can be directed to the corresponding author.

## Ethics Statement

The animal study was reviewed and approved by Comissão de Ética no Uso de Animais Universidade Federal do Paraná.

## Author Contributions

DG, MS, JC, and JCM performed the animal experiments. RM performed *in silico* analyses. VT-S, CS, JFM, and CC-O performed the conception and design of the work. VT-S and RM synthesized peptides. MS performed the assay qPCR for cytokines and parasites load. DG, VT-S, MS, GC, CS, and ER analyzed the data and wrote the draft of the manuscript. VT-S and CS corrected the final manuscript. VT-S supervised the entire project. All authors contributed to the article and approved the submitted version.

## Conflict of Interest

The authors declare that the research was conducted in the absence of any commercial or financial relationships that could be construed as a potential conflict of interest.
